# Technical Note: On the spatial correlation between robust CT‐ventilation methods and SPECT ventilation

**DOI:** 10.1002/mp.14511

**Published:** 2020-10-17

**Authors:** Edward Castillo, Richard Castillo, Yevgeniy Vinogradskiy, Girish Nair, Inga Grills, Thomas Guerrero, Craig Stevens

**Affiliations:** ^1^ Department of Radiation Oncology Beaumont Health Systems Royal Oak MI USA; ^2^ Department of Computational and Applied Mathematics Rice University Houston TX USA; ^3^ Department of Radiation Oncology Emory University Atlanta GA USA; ^4^ Department of Radiation Oncology University of Colorado Denver CO USA; ^5^ Department of Internal Medicine Beaumont Health Systems Royal Oak MI USA

**Keywords:** 4DCT, computed tomography, deformable image registration, SPECT, ventilation

## Abstract

**Purpose:**

The computed tomography (CT)‐derived ventilation imaging methodology employs deformable image registration (DIR) to recover respiratory motion‐induced volume changes from an inhale/exhale CT image pair, as a surrogate for ventilation. The Integrated Jacobian Formulation (IJF) and Mass Conserving Volume Change (MCVC) numerical methods for volume change estimation represent two classes of ventilation methods, namely transformation based and intensity (Hounsfield Unit) based, respectively. Both the IJF and MCVC methods utilize subregional volume change measurements that satisfy a specified uncertainty tolerance. In previous publications, the ventilation images resulting from this numerical strategy demonstrated robustness to DIR variations. However, the reduced measurement uncertainty comes at the expense of measurement resolution. The purpose of this study was to examine the spatial correlation between robust CT‐ventilation images and single photon emission CT‐ventilation (SPECT‐V).

**Methods:**

Previously described implementations of IJF and MCVC require the solution of a large scale, constrained linear least squares problem defined by a series of robust subregional volume change measurements. We introduce a simpler parameterized implementation that reduces the number of unknowns while increasing the number of data points in the resulting least squares problem. A parameter sweep of the measurement uncertainty tolerance, τ, was conducted using the 4DCT and SPECT‐V images acquired for 15 non‐small cell lung cancer patients prior to radiotherapy. For each test case, MCVC and IJF CT‐ventilation images were created for 30 different uncertainty parameter values, uniformly sampled from the range $\left[0.01,\;\;0.25\right]$. Voxel‐wise Spearman correlation between the SPECT‐V and the resulting CT‐ventilation images was computed.

**Results:**

The median correlations between MCVC and SPECT‐V ranged from 0.20 to 0.48 across the parameter sweep, while the median correlations for IJF and SPECT‐V ranged between 0.79 and 0.82. For the optimal IJF tolerance τ=0.07, the IJF and SPECT‐V correlations across all 15 test cases ranged between 0.12 and 0.90. For the optimal MCVC tolerance τ=0.03, the MCVC and SPECT‐V correlations across all 15 test cases ranged between −0.06 and 0.84.

**Conclusion:**

The reported correlations indicate that robust methods generate ventilation images that are spatially consistent with SPECT‐V, with the transformation‐based IJF method yielding higher correlations than those previously reported in the literature. For both methods, overall correlations were found to marginally vary for τ∈[0.03,0.15], indicating that the clinical utility of both methods is robust to both uncertainty tolerance and DIR solution.

## 1 INTRODUCTION

Computed tomography (CT)‐derived ventilation is an image processing modality that quantifies the apparent voxel volume changes within an inhale/exhale CT image pair as a surrogate for pulmonary ventilation.^1^, ^2^ With the exception of newer deep learning methods^3^ and methods based purely on image segmentation,^4^ there are two primary classes of CT‐ventilation methods: transformation based and intensity based. Both classes require, as preprocessing steps, lung volume delineation and deformable image registration (DIR). The DIR solution approximates respiratory motion and provides a spatial mapping between the inhale and exhale lung geometries. Intensity‐based methods estimate volume change using the Hounsfield Unit values of spatially corresponding inhale/exhale lung voxels.^2^, ^5^ Transformation‐based methods, in contrast, compute volume changes directly from the DIR‐defined spatial transformation via numerical approximation of the Jacobian Factor.^6^ While the images produced by transformation‐based methods have been shown to be highly variable with respect to numerical implementation,^7^ intensity‐based methods require key heuristic steps, including smoothing and vasculature segmentation. Taken together, these factors contribute to the suboptimal reproducibility of previously proposed CT‐ventilation methods.^8^, ^9^

We recently described two new CT‐ventilation algorithms designed to address reproducibility issues previously described in the literature^7^, ^8^, ^9^: the intensity‐based mass conserving volume change (MCVC) method^10^ and the transformation‐based Integrated Jacobian Formulation (IJF) method.^11^ As opposed to traditional approaches, MCVC and IJF estimate the Jacobian factor of the DIR transformation from a series of spatially corresponding lung subregions. The numerical uncertainty in the subregional volume change measurements is modeled with Gaussian statistics, which allows for their uncertainty to be characterized and, consequently, controlled through the definition of an uncertainty tolerance parameter. In this context, the uncertainty tolerance is defined with respect to the uncertainties associated with the DIR solution and does not address uncertainties associated with CT acquisition. This strategy provides robustness to DIR variability at the expense of measurement resolution. While IJF and MCVC images were both shown to be robust to DIR variations, no comparisons to clinically accepted modalities have been reported.

*The purpose of this study is to (a) introduce a parameterized numerical implementation of the MCVC and IJF methods and (b) characterize the spatial correlation between the resulting CT‐ventilation images and single photon emission computed tomography ventilation (SPECT‐V)*. Using a straightforward parameter sweep of the uncertainty tolerance, we examine the effect of the tolerance on the correlation between the resulting CT‐ventilation images and the SPECT ventilation images.

## 2 MATERIALS AND METHODS

### 2.A Subregional volume change estimation

Computed tomography‐ventilation methods employ DIR to compute a spatial transformation $\phi :R^{3}\to R^{3}$ which maps a reference image lung volume $\Omega^{\left(R\right)}$ onto a target image lung volume $\Omega^{\left(T\right)}$. For a general subregion $\Omega \in \Omega^{\left(R\right)}$, the volume scaling factor under ϕ is described by the Jacobian factor:

(1) $$\text{vol}\left(\phi (\Omega )\right)=\int_{\Omega }\det\left(J(x)\right)\;\mathrm{dx},$$

assuming ϕ is diffeomorphic. The transformation‐based IJF ventilation method^11^ numerically approximates Eq. (1) directly from the DIR solution as the sample mean of the membership oracle function

(2) $$\int_{\Omega }\det\left(J(x)\right)\;\mathrm{dx}\approx {〈f〉}_{\Omega }M=H,$$

where

(3) $$f\left(x;\;\Omega ,\phi \right)=\left\{\begin{array}{cc} 1, & \text{if}\;\phi^{‐1}\left(x\right)\in \Omega ,\\ 0, & \text{otherwise,}\; \end{array}\right)$$

and $M=\left|\Omega^{\left(T\right)}\right|$. Equation (2) is a “hit‐or‐miss” volume approximation^12^ and H represents the number of “hits” that land within Ω. The MCVC method,^10^ in contrast, represents an intensity‐based approach and requires the inhale/exhale CT image pair to be converted into two corresponding HU‐defined density functions, which we denote here as the reference image R(x), and the target image T(x). Assuming mass is conserved, the MCVC estimate is defined as

(4) $$\int_{\Omega }\det\left(J(x)\right)\;\mathrm{dx}\;\;\approx \;\frac{{〈R〉}_{\Omega }}{{〈T〉}_{\phi (\Omega )}}\cdot \left|\Omega \right|$$

where ${〈R〉}_{\Omega }$ and ${〈T〉}_{\phi (\Omega )}$ represent the sample mean densities within Ω and $\phi \left(\Omega \right)$, respectively. As previously described in detail, the uncertainties in the Eqs. (2) and (4) measurements can be characterized using the standard errors of the corresponding sample means.^10^, ^11^ For IJF, it was shown that with 95% probability

(5) $$\frac{\left|\text{vol}\left(\phi \left(\Omega \right)\right)\;\;‐H\right|}{H}\;\;\leq \;\;\tau ,\;\;\forall H\geq H^{*},$$

where the minimum hit count, $H^{*}$, depends on M and τ is a specified tolerance. For MCVC, it was shown that with 95% probability

(6) $$\left|\bar{R}‐{〈R〉}_{\Omega }\right|\leq \tau ,\;\;\text{if}\;\;\;\left|\Omega \right|\;\geq \;\frac{\beta^{2}}{\tau^{2}},$$

and

(7) $$\left|\bar{T}‐{〈T〉}_{\phi (\Omega )}\right|\leq \tau ,\;\;\text{if}\;\;\;H\;\geq \;\frac{\beta^{2}}{\tau^{2}},$$

for β=1.96. Equations (5), (6), (7) essentially state that the amount of uncertainty in the volume change estimates goes down as the size of Ω goes up. Thus, the improved robustness of MCVC and IJF comes at the expense of measurement resolution.

### 2.B Moving least squares ventilation image

A CT‐ventilation image, V(x), requires computing the discretized variables.

(8) $$V\left(x_{i}\right)=v_{i}=\det\left(J(x_{i})\right),\;\forall x_{i}\in \Omega^{\left(R\right)},$$

where $v_{i}\;>\;0$ represents the volume change of the undeformed unit volume voxel centered on $x_{i}$. Because both IJF and MCVC obtain volume change estimates for a series of subregions $\Omega_{k}\in \Omega^{\left(R\right)},\;\;k=1,2,...,K,$ their resulting mathematical formulations are equivalent. Specifically, for $|\Omega^{\left(R\right)}|\;=\;N$, the subdomain data acquisition process results in a linear system of equations that relates the unknown $v_{i}$ to the subdomain data:

(9) $$\begin{array}{c} Av=b,\;\\ A\in R^{K\times N},\;\;b\in R^{K\times 1},\;\;v\in R^{N\times 1}, \end{array}$$

where

(10) $$A_{\mathrm{ki}}=\left\{\begin{array}{cc} 1 & \text{if}\;x_{i}\in \Omega_{k}\\ 0 & \text{otherwise} \end{array}\right),$$

and the elements of b contain the corresponding subregional estimates [Eq. (2) for IJF and Eq. (4) for MCVC]. Previous IJF and MCVC implementations solve a computationally intensive, large‐scale linear least squares problems defined by Eq. (9). In order to facilitate broader accessibility of the established methods and improve overall method robustness, we instead parameterize V(x) with moving least squares (MLS) to reduce the number of unknowns and, consequently, the overall computational and memory storage requirements. This approach also allows for the definition of an overdetermined data fitting problem.

Given a set of L knot locations $z_{j}\in \Omega$ with corresponding scalar parameter values $q_{j}$, the Shepard's class of moving least squares approximation is defined as:

(11) $$V\left(x_{i};\;q\right)=v_{i}=\frac{\sum_{j=1}^{L}w\left(\left|\right|x_{i}‐z_{j}\left|\right|\right)\left(q_{j}\right)}{\sum_{j=1}^{L}w\left(\left|\right|x_{i}‐z_{j}\left|\right|\right)},$$

where the proximal weighting function is of the form

(12) $$w\left(r\right)=e^{‐\sigma r^{2}}.$$

The Eq. (11) parameterization reduces the number of unknowns required to generate the volume change (ventilation) image from N (the total number of voxels in the reference lung region of interest) down to L (the number of knots used for the discretization).

### 2.C Image Computation

A ventilation image, as defined by Eq. (11), can be recovered from the Eq. (9) subregional volume change estimates by solving the following constrained least squares problem:

(13) $$\begin{array}{c} \min_{q}{∥\hat{A}q‐b∥}^{2}+\sum_{k=1}^{L}{\left(q_{k}‐h/N\right)}^{2}\\ s\text{.t}.\;\sum_{i=1}^{N}v_{i}=h,\\ q_{i}\;\geq \;\varepsilon ,\;i=1,2,\ldots ,L, \end{array}$$

where

(14) $$\begin{array}{c} \hat{A}=AC,\\ \hat{A}\in R^{K\times L},\;C\in R^{N\times L}, \end{array}$$

and

(15) $$C_{\mathrm{ij}}=\frac{w\left(\left|\right|x_{i}‐z_{j}\left|\right|\right)}{\sum_{l=1}^{L}w\left(\left|\right|x_{i}‐z_{l}\left|\right|\right)}.$$

We point out that the data matrix $\hat{A}$ can be constructed without explicitly constructing A, thereby reducing the overall memory requirements.

The Eq. (13) inequality constraints prevent physically impermissible negative volumes. Requiring $q_{i}\;>\;0$ is enough to guarantee that the resulting $v_{i}$ (Jacobian estimates) are strictly positive since the Eq. (11) MLS parameterization is simply a moving average operator applied to the $q_{i}$. The equality constraint represents consistency with the global volume change, as measured on the full $\Omega^{\left(R\right)}$ and $\Omega^{\left(T\right)}$ lung volumes, which is often used as a validation metric for proposed ventilation methods.^1^, ^2^, ^5^, ^13^, ^14^ However, the constraint constant, h, depends on the choice of method. For transformation‐based IJF,

(16) $$h_{\mathrm{IJF}}=\left|\Omega^{\left(T\right)}\right|,$$

and for intensity‐based MCVC

(17) $$h_{\mathrm{MCVC}}=\frac{{〈R〉}_{\Omega^{\left(R\right)}}}{{〈T〉}_{\Omega^{\left(T\right)}}}\left(\left|\Omega^{\left(R\right)}\right|\right).$$

The Eq. (13) problem structure is equivalent to the one first presented in Ref. [11] with two exceptions. First, the number of subregional estimates can be chosen such that the problem is highly overdetermined N≥K≫L, while the reduced number of total unknowns allows for the application of standard solvers (as opposed to the customized augmented Lagrangian method presented in Refs. [10, 11]), including the MATLAB lsqlin interior‐point method (release R2019a, The Mathworks Inc, Natick, Massachusetts, USA). Second, whereas previous implementations regularized the problem by penalizing the norm of the spatial gradient of V, the proposed regularization model [second term in the Eq. (13) objective function] penalizes the variance of the moving least squares parameters with the mean defined as the average voxel volume change enforced by the equality constraint. This choice of regularization is derived from the recommendations of the AAPM Radiation Therapy Task Groupe No. 132, which state that large variations in the Jacobian are potentially indicative of error.^15^

The original approaches described in Refs. [10, 11] both defined an underdetermined data fitting matrix and relied on spatially smoothing regularization to ensure a well‐posed problem. This approach, when applied to the MLS parameterization, had the potential to induce over‐smoothing in the resulting images. In contrast, the Eq. (13) formulation defines an overdetermined data fitting matrix, $\hat{A}$, and penalizes overall variance in the MLS recovered function, as opposed to local smoothness which is already inherent to MLS functions.

### 2.D Numerical implementation

The maximum inhale and exhale phases from 4DCTs were used for this study. Lung masks were generated using a semi‐automated histogram segmentation (as done in Ref. [2]). A dart‐throwing algorithm^16^ was applied to $\Omega^{\left(R\right)}$ in order to generate MLS knot locations, $z_{j}\in \Omega^{\left(R\right)},$ with approximately 30 mm uniform spacing. Similar to the number of cubic spline knots used for lung CT DIR,^17^ this procedure results in approximately 250–300 knots. An additional point cloud was similarly acquired with approximately 7 mm uniform spacing to serve as the subdomain locations for Eq. (9). This resulted in approximately 20 000 to 30 000 subdomain measurement points. Each initial $\Omega_{k}$ subdomain was defined as a single voxel and then morphologically dilated with a 7 × 7 × 3 voxel structuring element until the tolerance criteria [Eq. (5) for IJF and Eqs. (6) and (7) for MCVC] were satisfied.

The spatial transformation $\phi^{‐1}$ is computed by applying the Quadratic Penalty DIR (QPDIR) algorithm to the inhale and exhale images. Briefly, QPDIR is an intensity‐based algorithm designed around a gradient‐free block coordinate descent strategy that essentially iterates between simple block matching and linear least squares operations to minimize the structural similarity index between an image pair. The implementation follows the description in Ref. [18], with the exception that an additional sum‐of‐squared difference term was included in the QPDIR objective function to improve lung mask alignment (as done in Ref. [19]).

Equation (13) is solved using the interior‐point method implemented in the MATLAB (release R2019a, The Mathworks Inc, Natick, Massachusetts, USA) optimization routine lsqlin.

### 2.E Image data

A validation of the MLS IJF and MCVC methods was conducted using the simulation (treatment planning) 4DCT and single photon emission tomography (SPECT) ventilation images for 15 NSCLC patients who received radiotherapy at our institution. Data were retrospectively evaluated according to an IRB approved study (IRB 2016‐037, clinicalTrials.gov #NCT02528942). Patients received definitive radiotherapy (defined as prescription doses of 45 to 75 Gy) and a planned concurrent chemotherapy regimen. The majority of enrolled patients had stage III disease. Single photon emission tomography imaging was acquired prior to delivery of the first radiotherapy fraction, ensuring no clinical lung function changes occurred due to treatment between the simulation 4DCT and SPECT acquisitions.

A Philips Brilliance Big Bore CT (version 3.6.7) with a bellows system was used for respiratory correlated imaging. The 4DCT images were acquired with x‐ray tube settings of 120 kVp and 599 mAs, and reconstructed using phase binning to produce an average CT image and 10 phase indexed CT images. The phase images were indexed from 0% to 90% in steps of 10% where 0% indicates full inhalation and 50% indicates full exhalation on the breathing curve. Final images were then exported into the DICOM (Digital Imaging and Communications in Medicine) standard (512 x 512 pixels per 2D slice image, voxel dimensions of 1.27 mm × 1.27 mm × 3 mm).

SPECT ventilation images were acquired on a dual head Siemens Symbia SPECT/CT scanner (Siemens Medical Solutions, USA), using a parallel hole, high‐resolution collimator and an energy window of 15% at a centerline of 140 keV. 1 mCi Tc99m diethylenetriaminepentaacetic acid aerosol inhalation was used for the ventilation scan. SPECT acquisition was performed in steps of 6° for the entire 360° of rotation, with a 25 s collection time for each step. Total scan acquisition time, for most patients, was under 30 min. Free breathing, attenuation‐corrected CT images were subsequently recorded with 130 kVp, and 75–100 mAs (weight dependent) during continuous tidal respiration. The final reconstructed SPECT ventilation images were then exported into DICOM (64 × 64 pixel per 2D slice image, voxel dimensions 6.00 mm × 6.00 mm × 2.00 mm).

### 2.F Uncertainty tolerance parameter sweep

A parameter sweep was conducted for both the MCVC and IJF methods in order to assess the effect of the uncertainty tolerance τ on the spatial correlation between SPECT ventilation and the resulting robust CT‐derived ventilation images. For each of the 15 test cases, a series of 30 MCVC and IJF CT‐ventilation images were computed using a uniformly sampled set of uncertainty tolerances ranging between τ∈[0.01,0.25] and converted to absolute volume difference^6^:

(18) $$\left|1‐V(x;\;q)\right|.$$

Each resulting image was spatially aligned with the SPECT ventilation image by first using affine registration to align the exhale 4DCT phase (on which the CT‐ventilation is computed) and the SPECT attenuation correction CT. The resulting affine transformation was then applied to the CT‐ventilation image and the voxel‐wise Spearman correlation was computed at the resolution of the SPECT ventilation, after applying a median filter with 3×3 structuring element to the SPECT image (as done in Ref. [20]).

## 3 RESULTS

**Fig. 1:**
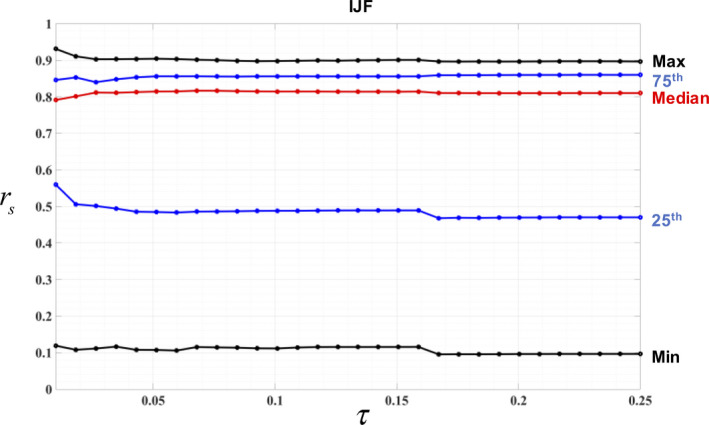
The maximum, 75th percentile, median, 25th percentile, and minimum Spearman correlations across all 15 cases as a function of the uncertainty parameter τ for the IJF method. The correlation values remain relatively constant across the parameter sweep. [Color figure can be viewed at wileyonlinelibrary.com]

**Fig. 2:**
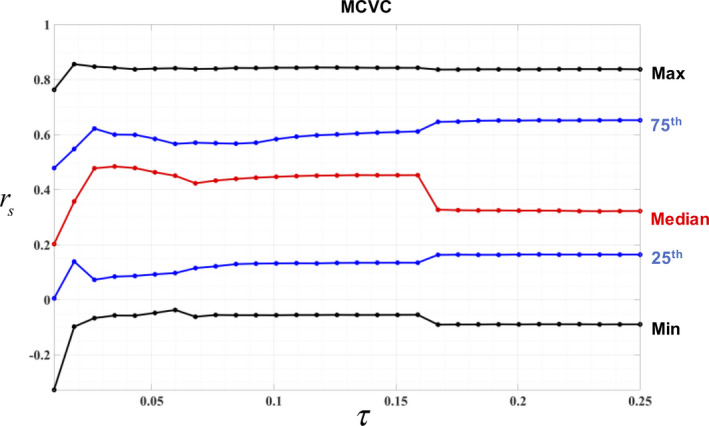
The maximum, 75th percentile, median, 25th percentile, and minimum Spearman correlations across all 15 cases as a function of the uncertainty parameter τ for the MCVC method. After variations for smaller τ, the correlation values remain relatively constant between 0.05 and 0.15. [Color figure can be viewed at wileyonlinelibrary.com]

The median Spearman correlations taken across all 15 test cases for each uncertainty parameter value are presented in Figs. 1 and 2. The median SPECT‐V and MCVC correlation values ranged between 0.20 and 0.48 across the parameter sweep, with the higher median values being achieved for τ∈[0.03,0.06]. The median SPECT‐V and IJF correlation values ranged between 0.79 and 0.82 across the parameter sweep and were relatively consistent, though the highest values were achieved for τ∈[0.05,0.15].

**Fig. 3:**
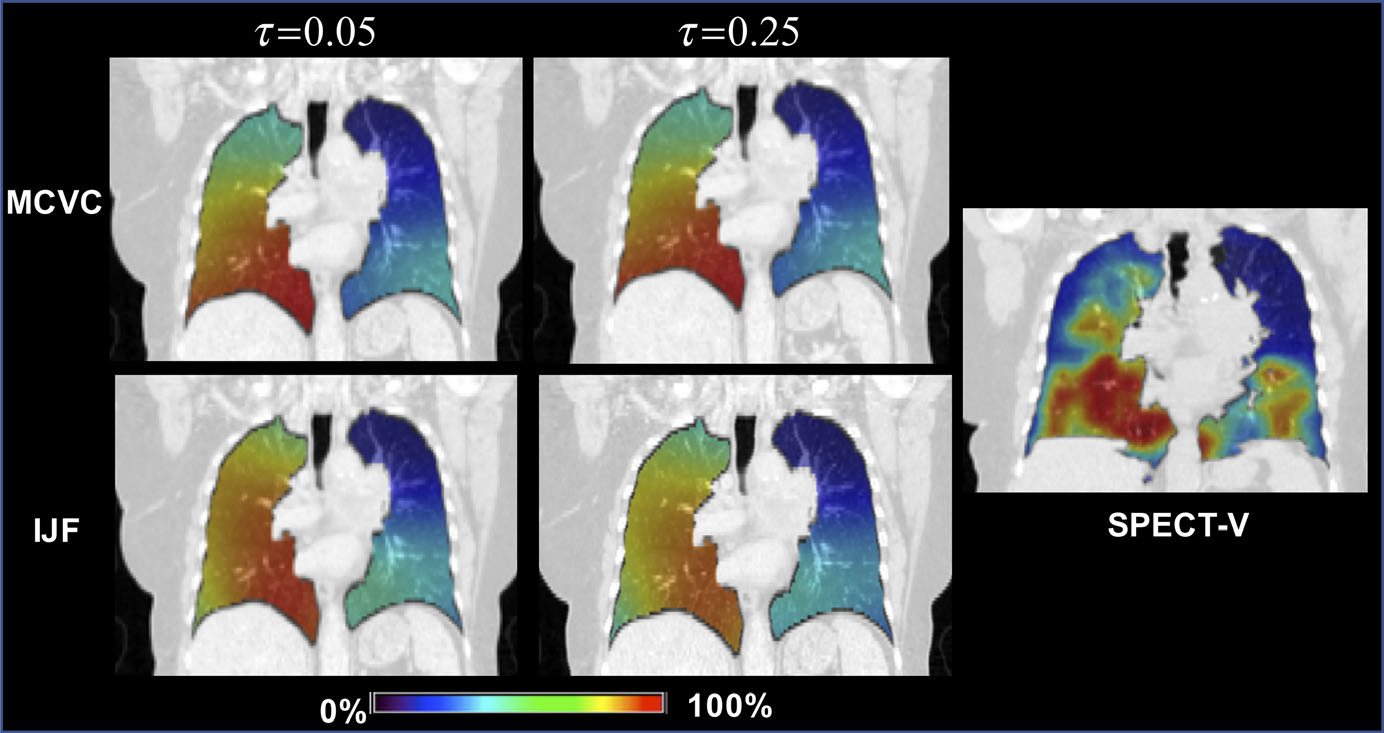
The MCVC (top row) and IJF (bottom row) ventilation images computed using τ=0.05 (left column) and τ=0.25 (right column) for the test case with the overall highest correlation (Case 9, Table I) with the corresponding SPECT‐V (far right). The images were converted to percentile images for direct visual comparison. All images denote decreased function in the left upper lung. The Spearman correlation between SPECT‐V and MCVC for τ=0.05 and τ=0.25 are 0.79 and 0.77, respectively. For IJF, the correlations are both 0.85.

As summarized in Table I, the IJF and SPECT‐V Spearman correlations across the 15 test cases for the optimal τ=0.07 ranged between 0.12 and 0.90. For MCVC, an optimal τ=0.03 generated Spearman correlations that ranged between −0.06 and 0.84 across the 15 test cases. Wilcoxon signed‐rank test applied to the optimal IJF and MCVC correlations in Table I indicate that the IJF method outperforms MCVC with high statistical significance (*P* = 0.0062). Figure 3 illustrates the case with overall highest correlation between the two methods (Case 9).

**Table I:** Spearman correlations for optimal τ.

Case #	1	2	3	4	5	6	7	8	9	10	11	12	13	14	15	Median
IJF τ = 0.07	0.12	0.86	0.73	0.90	0.87	0.64	0.82	0.79	0.84	0.44	0.83	0.90	0.37	0.25	0.82	0.82
MCVC τ = 0.03	0.05	0.48	0.62	0.10	0.75	0.50	0.52	0.08	0.77	0.84	−0.06	0.33	0.20	0.06	0.54	0.48

The Spearman correlations between SPECT ventilation and both IJF and MCVC across all 15 test cases are listed, using the optimal uncertainty tolerances, as determined by the parameter sweep (Figs. 1 and 2), for each method. In all cases, the correlation is significantly different from zero (*P* < 0.001).

## 4 DISCUSSION

Integrated Jacobian Method and MCVC represent a new class of robust CT‐ventilation methods that seek to reduce the uncertainty associated with DIR variability. Mass conserving volume change has the added benefit of not requiring the advanced pulmonary vasculature mask needed by previous intensity‐based methods.^2^ However, the previous numerical implementations of robust methods required the solution of a constrained, large scale, linear least squares problem defined by an underdetermined data fidelity term and a spatial smoothness inducing regularization. In this work, we introduce a parameterized variant of the original IJF and MCVC methods that allows for overdetermined data fitting and the use of common optimization solvers. In addition, robust CT‐ventilation methods require the definition of an uncertainty tolerance parameter. The uncertainty tolerance reflects the methodology's general property that robustness is gained at the expense of measurement resolution. As such, the parameter's selection represents the trade‐off between data fidelity and numerical stability. Previous work provided general bounds on parameters values that maintained DIR robustness. In this study, we characterize the effect the parameter has on the resulting correlation between robust CT‐ventilation and SPECT‐V.

Across the parameter sweep, the IJF method demonstrated less variability than MCVC. This reflects the inherent characteristics of intensity‐ and transformation‐based methods. Whereas, intensity‐based methods operate on nonhomogeneous image data, transformation‐based methods operate on the DIR displacement field, which is often optimized for spatial smoothness. This is the case for the QPDIR method utilized in this study.^18^ As indicated by the Fig. 1 results, accurate estimates for the subregional IJF sample means can, in practice, be acquired with fewer data points than required by the Eq. (5) bound. As such, the IJF median correlation appears to be more stable with respect to τ when compared to those of the MCVC method. Moreover, IJF demonstrated a significantly higher correlation with SPECT‐V than MCVC (*P* = 0.0062). This result is not altogether surprising considering the underlying mathematical formulations on which the two methods are based. Integrated Jacobian formulation recovers the DIR Jacobian values from the geometric information captured by the DIR solution. The MCVC method also approximates the Jacobian. But similar to other HU‐based methods, MCVC is derived under the assumption that the density variations observed between inhale and exhale CT are caused solely by changes in air content. This assumption is known to be invalid due to variations in pulmonary blood mass that occur throughout the breath cycle^21^ and is a potential source of error for MCVC.

The 0.48 median correlation generated by the optimal tolerance value for MCVC (Table I) across the 15 test cases is consistent with the results from the Ventilation and Medical Pulmonary Image Registration Evaluation (VAMPIRE) study, where the highest performing method achieved a median of 0.49.^20^ However, the 0.82 median correlation generated by IJF represents a significant improvement over previously reported results, though the comparison with VAMPIRE is not directly applicable since its analysis included Galligas PET and Xenon CT.^20^ Validation based on nuclear medicine imaging is in general challenging due to the lower spatial resolution and aerosol deposition artifacts common to SPECT ventilation. Future work includes conducting further validation with other functional imaging modalities, including PET Galligas, hyperpolarized gas MRI (as done in Refs. [22, 23]), and data from the VAMPIRE study.

**Fig. 4:**
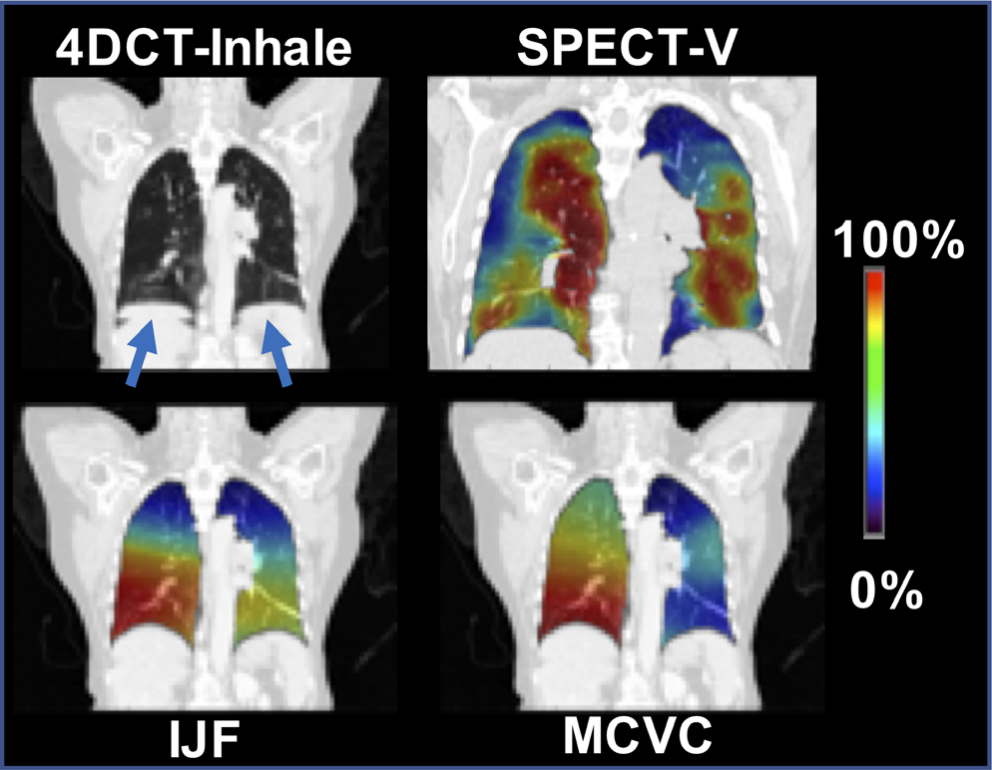
Top Row: The 4DCT‐Inhale phase (left) and SPECT (right) ventilation images for the case with the lowest over correlation in Table I (Case 1). Bottom Row: The IJF (left) and MCVC (right) superimposed on the 4DCT‐Exhale phase. Ventilation images were converted to percentile images for direct visual comparison. The inhale image possesses a phase‐bin artifact (blue arrows), which erroneously elevates the volume change signal in the lower right lung while erroneously suppressing it in the lower left. This leads to poor correlation with SPECT‐V.

The mathematical framework governing the IJF and MCVC methods guarantee that similar DIR solutions will generate similar ventilation images. This property was derived and demonstrated numerically in our previous studies.^10^, ^11^ Considering the correlations reported in Table I, robust ventilation algorithms provide a numerical framework that (a) is reproducible with respect to image processing pipeline and (b) generates results consistent with SPECT ventilation. However, the framework does not guard against 4DCT acquisition artifacts, which can lead to substantial differences between DIR solutions generated from competing methods. As illustrated in Fig. 4, phase‐binning 4DCT artifacts introduce erroneous geometric and intensity information into the Eq. (13) calculation, which can corrupt the resulting IJF and MCVC ventilation images. CT‐ventilation computed from breathhold CT acquisitions, which do not require phase binning, should therefore be expected to produce higher quality results. However, for 4DCT, an area of future work is in extending the IJF mathematical framework to be incorporated into both the DIR and segmentation algorithm, with the goal of reducing the effect of phase‐binning artifacts.

## 5 CONCLUSION

We have assessed the spatial correlation between SPECT‐V and two robust CT‐ventilation methods that are dependent on the definition of an uncertainty parameter τ. Results demonstrate that the proposed numerical implementation yields consistent results for uncertainty parameter values ranging between τ∈[0.03,0.15], with the highest correlations being achieved with τ=0.07 for IJF and τ=0.03 for MCVC. While MCVC demonstrated correlations consistent with those previously reported in the literature, the IJF method's SPECT‐V correlations represent a significant improvement over previous results, despite known issues with SPECT validation. Immediate future work involves comparisons with other ventilation modalities, such as PET Galligas and Hyperpolarized gas MRI, that are generally considered to be superior to SPECT.

## CONFLICT OF INTEREST

The authors have no conflict to disclose.

## References

[1] Guerrero T , Sanders K , Noyola‐Martinez J , et al. Quantification of regional ventilation from treatment planning CT. Int J Radiat Oncol Biol Phys. 2005;62:630–634. 15936537 10.1016/j.ijrobp.2005.03.023

[2] Castillo R , Castillo E , Martinez J , Guerrero T . Ventilation from four‐dimensional computed tomography: density versus Jacobian methods. Phys Med Biol. 2010;55:4661. 20671351 10.1088/0031-9155/55/16/004

[3] Zhong Y , Vinogradskiy Y , Chen L , et al. Technical note: deriving ventilation imaging from 4DCT by deep convolutional neural network. Med Phys. 2019;46:2323–2329. 30714159 10.1002/mp.13421 PMC7098066

[4] Kipritidis J , Hofman MS , Siva S , et al. Estimating lung ventilation directly from 4D CT Hounsfield unit values. Med Phys. 2016;43:33–43. 26745897 10.1118/1.4937599

[5] Guerrero T , Sanders K , Castillo E , et al. Dynamic ventilation imaging from four‐dimensional computed tomography. Phys Med Biol. 2006;51:777. 16467578 10.1088/0031-9155/51/4/002

[6] Reinhardt JM , Ding K , Cao K , Christensen GE , Hoffman EA , Bodas SV . Registration‐based estimates of local lung tissue expansion compared to xenon CT measures of specific ventilation. Med Image Anal. 2008;12:752–763. 18501665 10.1016/j.media.2008.03.007 PMC2692217

[7] Castillo E , Castillo R , Vinogradskiy Y , Guerrero T . The numerical stability of transformation‐based CT ventilation. Int J CARS. 2017;12:569–580. 10.1007/s11548-016-1509-x PMC5362676 28058533

[8] Du K , Bayouth JE , Ding K , Christensen GE , Cao K , Reinhardt JM . Reproducibility of intensity‐based estimates of lung ventilation. Med Phys. 2013;40:063504. 23718615 10.1118/1.4805106 PMC3676396

[9] Yamamoto T , Kabus S , von Berg J , et al. Reproducibility of four‐dimensional computed tomography‐based lung ventilation imaging. Acad Radiol. 2012;19:1554–1565. 22975070 10.1016/j.acra.2012.07.006 PMC5357435

[10] Castillo E , Vinogradskiy Y , Castillo R . Robust HU‐based CT‐ventilation from an integrated mass conservation formulation. Med Phys. 2019;46:5036–5046. 31514235 10.1002/mp.13817 PMC6842051

[11] Castillo E , Castillo R , Vinogradskiy Y , et al. Robust CT ventilation from the integral formulation of the Jacobian. Med Phys. 2019;45:2115–2125. 10.1002/mp.13453 PMC6510605 30779353

[12] Hammersley JM . Monte Carlo Methods , 1st edn. Dordrecht, Netherlands: Springer; 1964.

[13] Castillo R , Castillo E , McCurdy M , et al. Spatial correspondence of 4D CT ventilation and SPECT pulmonary perfusion defects in patients with malignant airway stenosis. Phys Med Biol. 2012;57:1855. 22411124 10.1088/0031-9155/57/7/1855

[14] Thomas G , Richard C , Kevin S , Roger P , Ritsuko K , Dianna C . Novel method to calculate pulmonary compliance images in rodents from computed tomography acquired at constant pressures. Phys Med Biol. 2006;51:1101. 16481680 10.1088/0031-9155/51/5/003

[15] Brock KK , Mutic S , McNutt TR , Li H , Kessler ML . Use of image registration and fusion algorithms and techniques in radiotherapy: report of the AAPM Radiation Therapy Committee Task Group No. 132. Med Phys. 2017;44:e43–e76. 28376237 10.1002/mp.12256

[16] Cline D , Jeschke S , White K , Razdan A , Wonka P . Dart throwing on surfaces. Comput Graph Forum. 2009;28:1217–1226.

[17] Delmon V , Rit S , Pinho R , Sarrut D . Registration of sliding objects using direction dependent B‐splines decomposition. Phys Med Biol. 2013;58:1303–1314. 23388109 10.1088/0031-9155/58/5/1303

[18] Castillo E . Quadratic penalty method for intensity‐based deformable image registration and 4DCT lung motion recovery. Med Phys. 2019;45:2194–2203. 10.1002/mp.13457 PMC6510611 30801729

[19] Rühaak J , Polzin T , Heldmann S , et al. Estimation of large motion in lung CT by integrating regularized keypoint correspondences into dense deformable registration. IEEE Trans Med Imaging. 2017;36:1746–1757. 28391192 10.1109/TMI.2017.2691259

[20] Kipritidis J , Tahir BA , Cazoulat G , et al. The VAMPIRE challenge: a multi‐institutional validation study of CT ventilation imaging. Med Phys. 2019;46:1198–1217. 30575051 10.1002/mp.13346 PMC6605778

[21] Myziuk N , Guerrero T , Sakthivel G , et al. Pulmonary blood mass dynamics on 4DCT during tidal breathing. Phys Med Biol. 2019;64:045014. 30654352 10.1088/1361-6560/aaff7b

[22] Tahir BA , Hughes PJC , Robinson SD , et al. Spatial Comparison of CT‐Based Surrogates of Lung Ventilation With Hyperpolarized Helium‐3 and Xenon‐129 Gas MRI in Patients Undergoing Radiation Therapy. (1879–355X (Electronic)). 10.1016/j.ijrobp.2018.04.077 30355463

[23] Mathew L , Wheatley A , Castillo R , et al. Hyperpolarized 3He magnetic resonance imaging: comparison with four‐dimensional x‐ray computed tomography imaging in lung cancer. Acad Radiol. 2012;19:1546–1553. 22999648 10.1016/j.acra.2012.08.007

